# Intrinsic photoisomerization dynamics of protonated Schiff-base retinal

**DOI:** 10.1038/s41467-019-09225-7

**Published:** 2019-03-14

**Authors:** Hjalte V. Kiefer, Elisabeth Gruber, Jeppe Langeland, Pavel A. Kusochek, Anastasia V. Bochenkova, Lars H. Andersen

**Affiliations:** 1 Department of Physics and Astronomy, Aarhus University, 8000 Aarhus C, Denmark; 2 Chemistry Department, Lomonosov Moscow State University, 119991 Moscow, Russia

## Abstract

The retinal protonated Schiff-base (RPSB) in its all-*trans* form is found in bacterial rhodopsins, whereas visual rhodopsin proteins host 11-*cis* RPSB. In both cases, photoexcitation initiates fast isomerization of the retinal chromophore, leading to proton transport, storage of chemical energy or signaling. It is an unsolved problem, to which degree this is due to protein interactions or intrinsic RPSB quantum properties. Here, we report on time-resolved action-spectroscopy studies, which show, that upon photoexcitation, *cis* isomers of RPSB have an almost barrierless fast 400 fs decay, whereas all-*trans* isomers exhibit a barrier-controlled slow 3 ps decay. Moreover, formation of the 11-*cis* isomer is greatly favored for all-*trans* RPSB when isolated. The very fast photoresponse of visual photoreceptors is thus directly related to intrinsic retinal properties, whereas bacterial rhodopsins tune the excited state potential-energy surface to lower the barrier for particular double-bond isomerization, thus changing both the timescale and specificity of the photoisomerization.

## Abstract (web summary)

The primary photoresponse of protonated Schiff-base retinal in visual and bacterial rhodopsins is fast sub-ps isomerisation. Here, the authors show that the fast photoisomerization of rhodopsin is related to an intrinsic retinal property, whereas bacterial rhodopsins tune the excited-state potential-energy surface and improve the isomerization timescale and specificity.

## Introduction

Bacteriorhodopsin (bR) is a seven helix membrane protein employed by halobacteria as a proton pump to convert light into chemical energy by transporting protons across the cell membrane^1,2^. It contains the all-*trans* retinal chromophore in the protonated Schiff-base form (RPSB), which isomerizes upon photoexcitation from its native all-*trans* to the 13-*cis* form with high specificity (64%) and speed (500 fs)^3–5^. According to the current belief, interactions within the protein facilitate isomerization that takes place with a speed about ten times faster than encountered without such interactions since isomerization takes about 2–10 ps in solutions^4,6,7^. In solutions, both 11-*cis* and 13-*cis* products are formed, with total quantum yields on the order of 10–30%^4,6,8,9^, and hence high specificity is obtained in proteins and not in solutions. It is also known that the retinal chromophore isomerizes upon photoexcitation in the gas phase; however, the efficiency as well as speed are unknown^10,11^.

Another class of retinal proteins is rhodopsins, responsible for the primary event in vision^12^, and having the 11-*cis* RPSB chromophore. Experimental^13–18^ and theoretical^19–21^ studies have shown that the 11-*cis* to all-*trans* isomerization in rhodopsins is extremely fast and likely caused by dynamics on an almost barrierless potential energy surface of the first (S_1_) excited state^22,23^. The progression from the initially populated Franck–Condon point to a fluorescent state in S_1_ takes, for example, <100 fs^16^, and full isomerization through a conical intersection is completed within 200 fs^13,14,19^. Rhodopsins also show high 65% quantum yields for isomerization to all-*trans*. The *cis* isomer is found to behave much faster than all-*trans* in MeOH at early times, but showed the same slow (4 ps) behavior on the longer timescale^24–26^. To account for both fast and slow dynamics, as well as the low quantum yield of only 0.22 in solution, it was proposed that a primary role of the protein is to adjust the ratio between ground-state chromophore conformations that lead to reactive and nonreactive decay channels^27^.

The two classes of proteins with all-*trans* and 11-*cis* RPSB chromophores both exhibit ultrafast sub-ps excited-state dynamics in their host proteins, yet their dynamics in solutions is multi-exponential and shows a long ps component. Interestingly, the lifetime of all-*trans* RPSB in solutions may be shortened by an order of magnitude from ~4 to ~400 fs upon chemical modifications of the retinal backbone with a wide spread in quantum yields^28,29^. The most likely explanation points towards tuning of the excited-state potential-energy surface around one or more conical intersections. These are notoriously hard to characterize theoretically, and electron correlation as well as nuclear dynamics must be treated accurately^30^. Based on QM/MM calculations of an RPSB model chromophore, it was proposed that the excited-state lifetime difference obtained in isolation and in MeOH may be traced to isomerization barriers primarily due to electrostatic interactions in the solution phase^23^.

Years of studies have been concerned with the related subject of spectral tuning (opsin shift) in proteins, where the band maximum traditionally has been compared to that obtained in MeOH solution (440 nm)^8^. This value is far from the intrinsic transition wavelength of about 610 nm obtained in the gas phase for both all-*trans* and 11-*cis* RPSB chromophores^31–33^, as well as those found in retinal-containing proteins. Interactions inside proteins, as well as in solutions, hence significantly change the energy gap between the ground and excited electronic states in the Franck–Condon region, and may also change the excited-state potential-energy surface around conical intersections. This may cause optimum conditions for fast isomerization and high quantum yields in proteins. Alternatively, the difference may be ascribed to significant perturbations taking place primarily in solutions, which slow down the isomerization and change the quantum yield significantly.

Despite numerous and advanced time-resolved studies of retinal proteins and RPSB chromophores in solutions, reference measurements obtained in the gas phase without solvent effects (H-bonding, steric interactions, electric fields, counterions, etc.) have not been obtained. Such measurements are needed to access the exact role of a hosting medium with respect to key properties such as quantum yields and excited-state lifetimes, which in turn are also related to fluorescence properties^25,34,35^. The reason for this situation is the tremendous experimental challenge encountered when working with extremely dilute clouds of ions in the gas phase, where the chromophore density is orders of magnitude smaller than in solutions. Despite the challenges, such data may provide a stringent test of ab initio theories, which must be able to satisfactorily deal with the chromophore in isolation before any predictions of transition energies, lifetimes, and quantum yields in environments may be considered fully reliable.

Here, we report on an experimental excited-state dynamics study of the RPSB chromophores in isolation (gas phase). A new experimental approach, combining time-resolved action spectroscopy^36^ with femtosecond pump-probe techniques^37^ in an ion-storage ring, is used. The new technique is applicable to anions^36^ as well as cations, such as the retinal protonated Schiff-base chromophore. The studies include variations of key parameters, such as the pump and probe wavelengths and the chromophore-ion temperature. High-level ab initio calculations are performed to support our conclusions.

## Results

### Pump-probe measurements

**Fig. 1:**
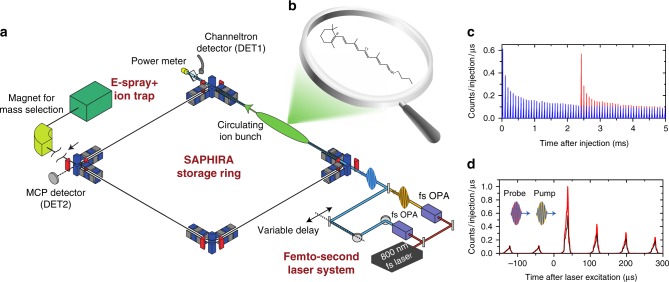
Experimental technique. **a** The experimental setup at the electrostatic ion-storage ring SAPHIRA^38^. Ions are produced by electrospraying retinal chromphores dissolved in methanol. The chromophore ions are stored in SAPHIRA before being irradiated by femtosecond laser pulses. **b** The all-*trans* protonated Schiff-base retinal chromophore. **c** Circulating ions produce neutral fragments from collisions with the residual gas (blue curve). Absorption of photons (after 2.4 ms of ion storage) increases the internal energy and causes statistical fragmentation after internal conversion to the ground state, as measured by the micro-channel plate (MCP) detector (red curve)^39^. **d** At each pump-probe delay laser-induced fragmentation is registered as a function of storage time in SAPHIRA. The pump-probe signal is obtained by subtracting background contributions (collisional background as well as single-pulse events) from the total two-pulse signal (see Supplementary Figure 1)

The experiments are performed at the ion-storage ring SAPHIRA^38^ (Fig. 1a), where RPSB chromophore ions (Fig. 1b) are stored in vacuo for several milliseconds. The absorption of photons is registered by the induced fragmentation and subsequent detection of neutral photofragments (Fig. 1c, d). The rate of statistical fragmentation (action) depends on the total internal energy of the ions, and therefore on the number of photons absorbed^39^. From the fragmentation rate, we are able to deduce when, after initial S_0_ → S_1_ excitation, a second probe photon of given energy and delay may be absorbed. This has been utilized in two different pump-probe schemes to obtain the excited-state decay as well as the ground-state recovery.

**Fig. 2:**
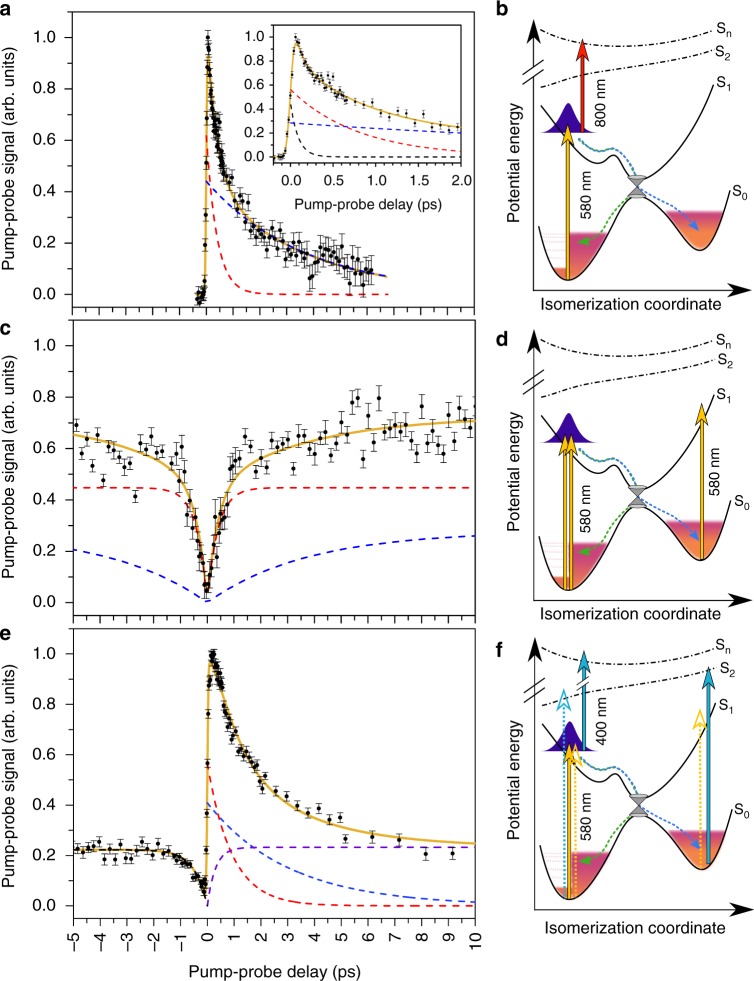
Pump-probe measurements of retinal protonated Schiff-base (RPSB) in vacuo. **a**, **b** Excited-state decay seen as the two-photon, pump-probe signal as a function of delay between pump (580 nm) and probe (800 nm) pulses. The full curve is from a fit with two exponential functions folded with the 80 fs cross-correlation in the experiment. The dashed lines represent the two decay components in the fit (395±42 fs and 3.7±0.4 ps). The insert shows a fit with three lifetime components, which specifically takes into account the separate early sub ~100 fs dynamics (fitted lifetimes 70±20 fs, 785±185 fs, and 5.6±1.8 ps). Data with 900 nm probe and other pump wavelengths within the S_0_→ S_1_ absorption band may be found in the Supplementary Table 1. **c**, **d** Ground-state recovery revealed by the two-photon, pump-probe signal as a function of delay between pump (580 nm) and probe (580 nm) pulses. The full curve is a fit with the two lifetimes found from the decay of the excited state (shown in **a**). **e**, **f** Dynamics revealed by the two-photon, pump-probe signal as a function of delay between pump (580 nm) and probe (400 nm) pulses. The chromophore may here absorb two photons (pump and probe) at all times, except right after excitation to S_2_ by 400 nm at small negative pump-probe delays. The fast recovery on the negative time axis is associated with the return to S_0_ after initial excitation (400 nm) to S_2_. All data shown here are recorded at room temperature. All error bars presented are one standard deviation (SD)

The present new approach of two-dimensional time-resolved action spectroscopy works as follows. A pump pulse first excites the RPSB chromophore in the broad S_0_ → S_1_ absorption band, which in the gas phase is between 550 and 650 nm^31–33^. To follow the evolution in the *excited state*, a probe wavelength of either 800 or 900 nm was used. At these wavelengths, subsequent S_1_ → S_*n*_ excitation may take place^33^ causing fast statistical fragmentation of the chromophore ions after sequential absorption of two photons (pump and probe) and internal conversion to the S_0_ ground state. The ground state is not re-excited at these probe wavelengths, and hence the fast two-photon fragmentation signal disappears as soon as the system has left the S_1_ excited state and returned to S_0_ (Fig. 2a, b). In contrast, when a probe wavelength, resonant with the S_0_ → S_1_ electronic transition is used, one retrieves the *ground-state* recovery after internal conversion by the growing fast, two-photon action signal with increasing pump-probe delay (Fig. 2c, d). Identification of the absorption of two consecutive photons is provided by the time at which the molecular dissociation takes place, hence time in two dimensions: the statistical fragmentation time and the pump-probe delay.

The time-resolved pump-probe fragmentation signal with 580 nm pump pulses and 800 nm probe pulses is shown in Fig. 2a. A fast decay is observed when the 580 nm pump comes prior to the 800 nm probe (positive delay). Here the RPSB chromophore is first excited to S_1_ by the pump pulse, and further excited to S_3_ by the 800 nm probe pulse^33^. No fast, two-photon, action is observed when the pulses arrive in the reversed order (negative delay) due to the lack of ground-state excitation with 800 nm pulses. The signal also disappears at late times when the ground state is repopulated after internal conversion. The data show two lifetimes of approximately 400 fs and 3 ps, with an ultrafast sub-100fs component, revealed from a three-exponential fit (Fig. 2a inset), ascribed to wavepacket dynamics in the Franck–Condon region. Similarly, when the probe-pulse wavelength is set to probe the *same* S_0_ to S_1_ transition as the pump pulse (580 nm, Fig. 2c), the two-photon absorption signal appears when the S_0_ ground state becomes repopulated with the same set of lifetimes. The two lifetimes were found to be insensitive to the choice of wavelength (see Supplementary Figures 2 and 3).

In Fig. 2e, we consider the case with 580 and 400 nm pulses. Both are capable of exciting the ground state (to S_1_ and S_2_, respectively), though with different efficiency. This gives a signal at asymptotic negative as well as positive delay times. The S_0_ → S_2_ transition has a low oscillator strength, and the 400 nm probe pulse is absorbed from the S_1_ state more efficiently than from S_0_ after internal conversion^33^, resulting in the overall decay of the signal at positive delay times. Notably, when the RPSB is excited to S_2_ by 400 nm excitation, it returns to the ground state in ca. 500 fs, as evidenced from the dynamics at negative delay times. Such fast relaxation of RPSB in S_2_ was also seen in fluorescence studies of RPSB ions in solvents^35^. However, more detailed gas-phase studies are required to fully resolve the relaxation dynamics of RPSB following its excitation to S_2_.

**Fig. 3:**
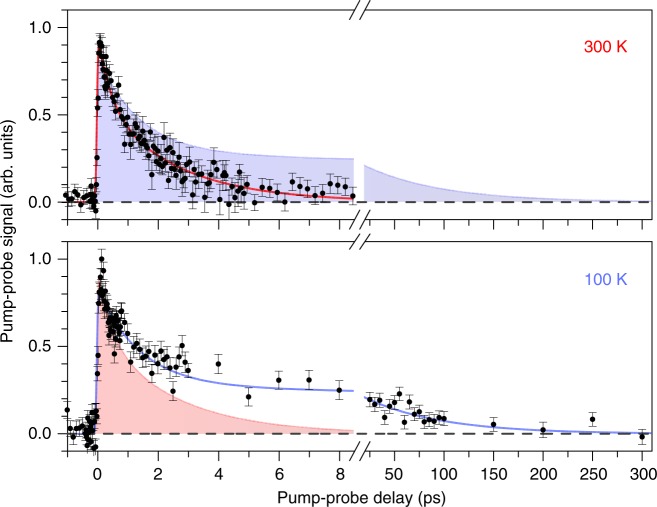
Pump-probe signal as a function of the pump-probe delay at 300 and 100 K. The excited-state decay of retinal protonated Schiff-base (RPSB) at two temperatures. Upper graph: Two-photon signal as a function of delay between pump (600 nm) and probe (800 nm) pulses, recorded at 300 K. Lifetimes of about 400 fs and 3 ps are here deduced. Lower graph: Same as upper graph, but recorded at 100 K where the lifetimes are 1.4 and 77 ps. The shaded areas are shown to provide a cross-comparison with the data obtained at the other temperature. All error bars presented are one standard deviation (SD)

The temperature of the chromophore ions prior to injection into the ion-storage ring may be controlled by cooling the ion trap inside the ion source with liquid nitrogen. At 100 K, the lifetimes are found to increase significantly to 1.4 and 77 ps, as shown in Fig. 3 (compared to 400 fs and 3 ps at 300 K). This indicates that both lifetime components are controlled by one or more barriers in the excited state.

It is in general challenging to completely isolate all-*trans* from single-*cis* isomers of RPSB in the gas phase as well as in solutions, where exposure to light generates isomerized forms that are non-statistically populated^32,40,41^. Ion mobility measurements of the RPSB chromophore have shown an almost equal content of all-*trans* versus single *cis* isomers after electrospraying the all-*trans* isomer^41^. Based on these results, we might expect a significant fraction of the ions populated in single *cis* isomers, separated from the all-*trans* configuration by relatively high barriers. We therefore consider both all-*trans* and single *cis* isomers as relevant RPSB forms in the following discussion.

### High-level ab initio calculations

**Fig. 4:**
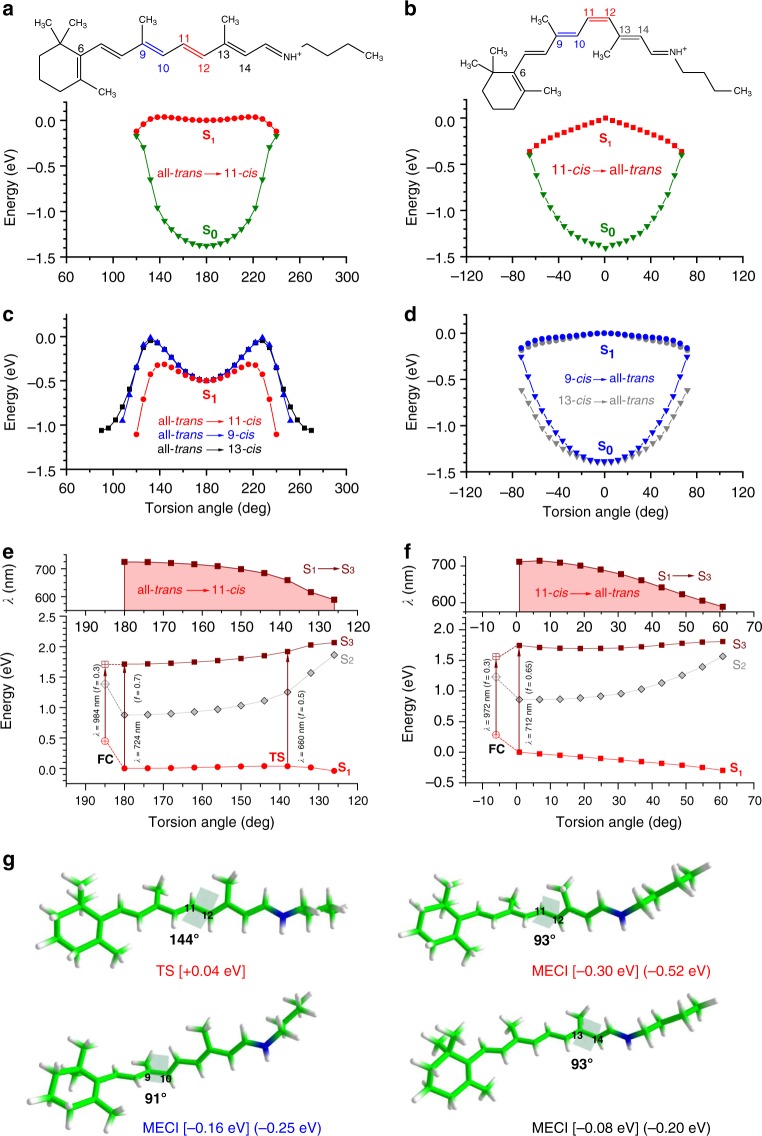
Calculated potential-energy curves along the isomerization coordinates in all-*trans*, 11-*cis*, 9-*cis*, and 13-*cis* retinal protonated Schiff-base (RPSB). **a**–**d** Excited-state relaxed geometry scans for rotation about double bonds are shown in red (∠C_10_C_11_ = C_12_C_13_), blue (∠C_8_C_9_ = C_10_C_11_), and black (∠C_12_C_13_ = C_14_C_15_). Ground-state energies calculated at the S_1_ optimized geometries along the lowest-energy pathway are shown in green. The planar structures in S_1_ are obtained through the unconstrained optimization for all isomers, except for the 11-*cis* isomer, where the torsion angle is kept fixed. Note that the 6s-*cis* and 6s-*trans* rotomers are found to have the same barrier heights that hinder isomerization in S_1_ in all-*trans* RPSB (see Supplementary Figure 4). **e**, **f** Transient absorption calculated as vertical excitation energies S_1_–S_3_ in all-*trans* and 11-*cis* RPSB. Oscillator strengths *f* along the reaction pathways are shown in brackets. Note that the S_1_–S_2_ transition has a lower oscillator strength of <0.1 and gains some intensity (*f* = 0.2) through mixing with the S_3_ state near the Franck–Condon (FC) point and closer to the S_1_/S_0_ conical intersections. **g** Optimized structures of the lowest-energy transition state in S_1_ in all-*trans* RPSB (TS) and the minimum-energy conical intersections S_1_/S_0_ (MECI). Carbon atoms are depicted in green, hydrogen atoms in white, and the nitrogen atom in blue. Energies in square and round brackets are shown with respect to the all-*trans* and the corresponding single *cis* RPSB planar structures in S_1_, respectively

To interpret the experimental results, we have performed multi-state multi-reference perturbation theory excited-state calculations for the all-*trans* and single *cis* isomers of RPSB, using the XMCQDPT2^42^/SA(2)-CASSCF(12/12)/cc-pVDZ method. In Fig. 4a–d, S_1_ relaxed geometry scans for rotation about three central double bonds in the all-*trans* and single *cis* chromophores are shown. We found that all-*trans* RPSB has small barriers that hinder rotation about the C_9_ = C_10_, C_11_ = C_12_, and C_13_ = C_14_ bonds and specifically that the barrier is the smallest (by a factor of 2.5) for rotation about the C_11_ = C_12_ bond. For all-*trans* → 11-*cis* isomerization, the barrier is 0.04 eV and the corresponding minimum-energy conical intersection (MECI) lies 0.3 eV below the minimum in S_1_, thus providing a direct route to the ground state after crossing the barrier in S_1_. The associated excited-state lifetime of all-*trans* RPSB is calculated to be 4 ps at 300 K and as long as 92 ps at 100 K. These values are in agreement with the long time component observed in the present measurements, 3 ps at 300 K and 77 ps at 100 K, which is thus assigned to the decay of S_1_ in all-*trans* RPSB.

According to our calculations, the 9-*cis*, 11-*cis*, and 13-*cis* isomers exhibit essentially flat potential-energy surfaces around the corresponding minima in S_1_ and hence undergo ultrafast, almost barrierless internal conversion from S_1_ to S_0_. The steepest pathway is found for the 11-*cis* isomer (Fig. 4b). This is consistent with our measured fast sub-ps component. However, this component also shows a temperature dependence and increases from 400 fs at 300 K to 1.4 ps at 100 K (Fig. 3). Using the experimentally determined lifetime at the low temperature, we can estimate a barrier height for the *cis* isomers in S_1_. This gives a barrier which is approximately one order of magnitude lower than that found in all-*trans* RPSB. Such a small barrier height is consistent with the calculated flat potential-energy surface of the S_1_ state for the *cis* isomers, in particular for 9-*cis* and 13-*cis* RPSB. The almost barrierless photoisomerization in the gas phase documented here is indeed in agreement with CASSCF theory for the 11-*cis* isomer^21^.

**Fig. 5:**
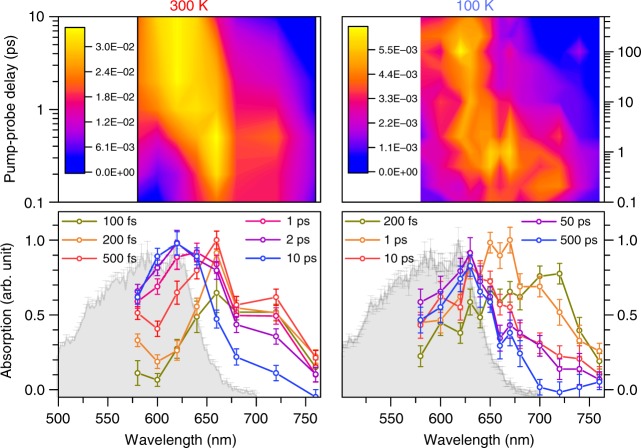
Transient absorption (TA) measurements of retinal protonated Schiff-base (RPSB) in the gas phase at 300 and 100 K. The TA data are shown as a function of variable probe wavelength (fixed 600 nm pump). The grey area shows the ground-state absorption profile at 300 K (one-photon spectrum). The transient absorption spectra at asymptotic long times (10–500 ps) correspond to absorption from a hot ground state, peaking at 620 nm. The excited-state absorption in the red part disappears with time concomitantly with a rise of the ground-state absorption with a lifetime, which strongly depends on temperature. Three major peaks at 720, 660, and 620 nm are seen at both temperatures. See discussion in the text

The calculated S_1_–S_*n*_ vertical excitation energies along the C_11_ = C_12_ isomerization coordinate of all-*trans* and 11-*cis* RPSB (Fig. 4e, f) show that the transient absorption (TA) corresponds to the S_1_–S_3_ excitation, which is the brightest transition along the entire reaction pathway. It shifts to shorter wavelengths as a vibrational wavepacket travels in the S_1_ state towards the conical intersection (Fig. 4g). Indeed, the enhanced experimental TA signals, seen at 720 and 660 nm, match the bright S_1_–S_3_ vertical transitions at the optimized geometries of the S_1_ minimum and transition state, respectively (see Fig. 5).

The S_2_ state does not appreciably mix with S_1_ along the reaction pathway as also shown by other methods like CASSCF^21^. The smallest energy gap is 0.9 eV and steeply rising as the chromophore twists (see Fig. 4e, f). The mechanism of internal conversion is thus defined solely by the interplay between the ground and first excited states in RPSB, where the latter is dominated by a charge-transfer character, as also demonstrated recently for RPSB in the gas phase^43^.

The absence of a potential-energy barrier in the single *cis* isomers can be attributed to sterical hindrance imposed by the repulsive interaction of the methyl groups with the hydrogen atoms of the conjugated tail of the chromophore, resulting in destabilization of the planar equilibrium structures in S_1_ and almost barrierless internal conversion in *cis* isomers. The equilibrium structures of 9-*cis* and 13-*cis* RPSB lie 0.09 and 0.12 eV higher in energy compared to that of all-*trans* RPSB, respectively. In the 11-*cis* isomer, the S_1_ planar structure lies 0.22 eV higher in energy compared to that of all-*trans* RPSB and is not stable with respect to rotation about the C_11_ = C_12_ bond at the XMCQDPT2/SA(2)-CASSCF(12,12)/cc-pVDZ level of theory.

The fraction of ions in the short-lived component increases from 40 to 57% in going from 300 to 100 K, thus indicating that the *cis* isomers are most likely formed through light exposure or collisions. The thermal backward *cis* → *trans* reaction in the ground state is strongly influenced by the temperature of the ions, hence resulting in a larger fraction of the *cis* isomers trapped in the ground state at low temperature. These fractions are also consistent with ion mobility spectrometry (IMS) measurements^32,41^. Furthermore, the all-*trans* → *cis* photoreaction shows specificity in the gas phase, as evidenced by our calculations, thus predominantly resulting in the 11-*cis* isomer.

**Fig. 6:**
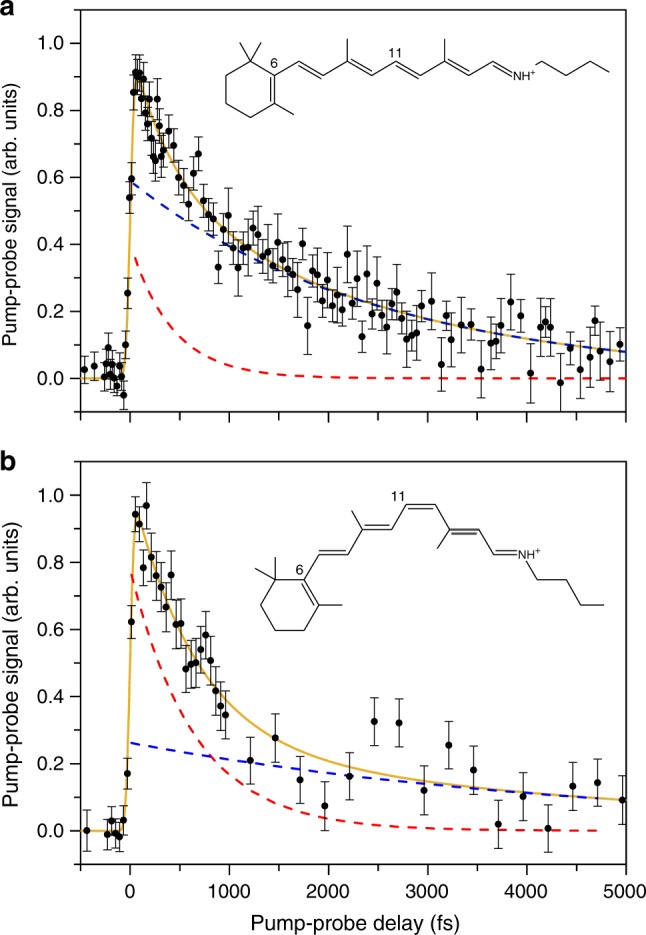
Pump-probe signal for all-*trans* and 11-*cis* retinal protonated Schiff-base (RPSB) samples in the gas phase. Pump-probe signal for all-*trans* (top) and 11-*cis* (bottom) samples obtained with 600 nm pump and 800 nm probe at room temperature. The full curve is from a fit with two exponential functions folded with the 80 fs cross-correlation in the experiment. The dashed lines represent the two decay components in the fit 424 ± 121 fs and 2.5 ± 0.3 ps for all-*trans* and 650 ± 160 fs and 4.7 ± 3 ps for 11-*cis* RPSB. All error bars presented are one standard deviation (SD)

Starting with an 11-*cis* sample, the decay is also described by two components, a short 650 ± 160 fs component and a long 4.7 ± 3 ps component at 300 K (see Fig. 6), similar to the dynamics of the all-*trans* sample. Here, the fraction of ions in the short-lived component is 78% (300 K), supporting that the fast component is connected with the 11-*cis* isomer.

The excited-state relaxation dynamics of a recently discovered bacterial rhodopsin, KR2, which functions as a light-driven sodium-ion pump and contains all-*trans* RPSB shows several time constants^44^. A major 180 fs component is attributed to the reactive decay of S_1_, while two minor components of 3 and 30 ps are assigned to nonreactive S_1_ states. The decay by isomerization of S_1_ occurs three times faster in KR2 than in bR, and hence the protein environment can indeed change the timescale of photoisomerization.

The mechanism that produces nonreactive S_1_ states, also reported for other retinal-containing proteins^44^, is not clear. However, in light of our findings, it is tempting to assign the slow picosecond components observed in KR2 to the dynamics of all-*trans* RPSB in its native, planar conformation inside the protein, assuming that active-site structural heterogeneity involves different conformations of all-*trans* RPSB inside the chromophore-binding pocket. The protein may facilitate photoisomerization by changing the native conformation of all-*trans* RPSB through pre-twisting it in S_0_, thus reducing the barrier height for double-bond rotation in S_1_ and decreasing the timescale considerably. The slow 3 ps component found in KR2 is remarkably similar to that observed in the present study for the bare all-*trans* RPSB. Moreover, our calculated lifetimes for isomerization about C_11_ = C_12_ and C_13_ = C_14_ (or C_9_ = C_10_) bonds are ~5 and ~40 ps, respectively. These values are to be compared with the slow 3 and 30 ps time constants of KR2. In both cases, the planar all-*trans* PSBR chromophore is slow and most likely not operational in the photocycle of the protein. Hence, the native planar conformation of the chromophore might be responsible for slow nonreactive dynamics in retinal-containing proteins.

## Discussion

The excited-state dynamics of RPSB-containing proteins as well as of RPSB in solutions shows a variety of time constants, typically in the sub-ps and few-ps regime. We have studied, for the first time, the dynamics of the bare RPSB chromophore in vacuo and found timescales of the very same order. When excited to the first electronically excited state, the decay is found to be tri-exponential, where the two short lifetimes together are represented by a ~400 fs decay. This decay time is independent of the excitation wavelength, but temperature dependent, raising by a factor of about three when the RPSB chromophore is cooled to 100 K. It gives evidence that a barrier as small as 30 cm^−1^ is operative in internal conversion for the bare chromophore. A longer, several picosecond component is also involved. This component reaches a decay time of almost 100 ps when cooled to 100 K. According to our calculations this is consistent with a 0.04 eV (~300 cm^−1^) energy barrier in S_1_ for all-*trans* RPSB. Experimentally and theoretically the long picosecond decay is thus assigned to RPSB in the all-*trans* form. Theoretically, we find the isomerization of RPSB in the 11-*cis* form to be essentially barrierless in S_1_ and the short sub-picosecond component may therefore be ascribed to 11-*cis* RPSB. Based on our findings, we conclude that the visual photoreceptors rely on the ultrafast intrinsic photoresponse of their 11-*cis* chromophore, whereas bacterial rhodopsins tune the photoresponse of the all-*trans* RPSB chromophore by particularly reducing the barrier height about the C_13_ = C_14_ double bond, thus changing both the timescale and specificity of photoisomerization of RPSB compared to those found in the intrinsic photoresponse, where the lowest barrier is for rotation about the C_11_ = C_12_ double bond.

## Methods

### Experimental setup

The experiments were performed at the SAPHIRA electrostatic ion-storage ring^38,45^, where an electrospray ion source with a multipole ion trap was used to produce bunches of bio-chromophores in vacuo.

### Ion source and sample preparation

All-*trans* as well as 11-*cis* Schiff-base retinal chromophores were dissolved in methanol and electrosprayed as protonated ions from a steel needle. Solvent molecules were evaporated off in the capillary, which was heated to about 100 °C, resulting in bare, singly charged molecules, which were transported through an octopole ion guide to a 16-pole radio frequency ion trap. Helium buffer-gas collisions reduced the translational kinetic energy causing the ions to be trapped. The ions were thermalized to the buffer-gas temperature (100 or 300 K) while trapped. At 100 Hz repetition rate, ions were extracted as 20 μs bunches. The ion source was located on a 4 kV high-voltage platform, which provided extraction from the source and subsequent acceleration to 4 keV. The retinal ions were mass-to-charge selected through a dipole magnet prior to injection into SAPHIRA.

### Ion storage and detection

SAPHIRA has four quadrupole 90° bending electrodes, mounted with quadrupole triplet lenses at the entrance and exit of each corner to keep the ion beam collimated in the ring. Two particle detectors mounted at the end of corner 3 and 4 detected neutrals generated by residual gas collisions (background pressure 2.0E−8 mbar and from photo-induced dissociation, see Supplementary Figure 1).

### Laser system and optical setup

Laser pulses were generated by a Ti:Sapphire-based Libra HE-UFS oscillator and regenerative amplifier system, delivering 4 mJ, 50 fs pulses with 1 kHz repetition rate. The pulses were split 3:1 to separately pump two TOPAS-C optical parametric amplifiers, which generated the pump and probe pulses. One of the beams was delayed by a motorized delay stage. The two beams were collinearly overlapped using a beam splitter or dichroic mirror, depending on the pump and probe wavelength. The cross-correlation of two 580 nm pulses was measured to be 80 fs in a β-barium borate crystal by sum-frequency generation. The pulse energies were 100 μJ at 580 nm, 60 μJ at 610 nm, and 200 μJ at 400 nm.

### Typical experimental cycle

The ion bunches circulated for about 2 ms in SAPHIRA prior to laser excitation. The first laser pulse excited the retinal chromophores from the S_0_ ground state to the S_1_ excited state. Internal conversion brought the ions back into the ground state, now with significantly increased internal energy, leading to fragmentation on the millisecond timescale. The total pump-probe signal appears from the increased fragmentation of both pump and probe photon absorption. For the final data analysis, we consider only the counts of the first pump-probe signal peak (as a function of the pump-probe delay), to limit statistical fluctuations from background subtraction. Error bars are estimated considering only the statistics of the neutral counts.

### Computational methods

All energy and gradient calculations in both excited and ground states are performed using the extended multiconfiguration quasi-degenerate perturbation theory, XMCQDPT2^42^. Structures of the stationary points in S_1_ and the S_1_/S_0_ MECIs, as well as relaxed geometry potential-energy scans along isomerization coordinates in S_1_ are obtained at the XMCQDPT2^2^/SA(2)-CASSCF(12,12)/cc-pVDZ level of theory. Vertical excitation energies S_1_–S_n_ along reaction pathways are calculated at the XMCQDPT2[7]/SA(7)-CASSCF(12,12)/cc-pVDZ level of theory. The Firefly package^46^, version 8.2.0, is used for all electronic structure calculations. Excited-state lifetimes of all-*trans* RPSB are calculated using transition state theory as an inverse of a sum of rate constants that refer to rotation about three double bonds. We assume the same thermal population of vibrational levels in S_1_ as in S_0_, following excitation that is close to the adiabatic transition in RPSB. An excess of vibrational energy gained upon excitation is therefore considered to be small compared to an average vibrational energy of the chromophore ions equilibrated in the ground state prior to excitation. Partition functions for the reaction modes are calculated based on the exact eigenvalues found as numerical solutions to the one-dimensional Schrödinger equation using the calculated XMCQDPT2 potentials that hinder rotation about double bonds in S_1_.

## Supplementary information

Supplementary Info

## Data Availability

The authors declare that the data supporting the findings of this study are available from the corresponding author upon reasonable request.
