# Supplementary material for: Intrinsic photoisomerization dynamics of protonated Schiff-base retinal
Source: Nat Commun. 2019 Mar 14;10:1210. doi: 10.1038/s41467-019-09225-7 (PMC6418104; doi:10.1038/s41467-019-09225-7)
Supplement: Supplementary file 1 — Supplementary Info [file 41467_2019_9225_MOESM1_ESM.pdf]

Supplementary Information

Intrinsic photoisomerization dynamics of protonated Schiff-base retinal

Hjalte V. Kiefer, Elisabeth Gruber, Jeppe Langeland, Pavel A. Kusochek, Anastasia V. Bochenkova and Lars H. Andersen

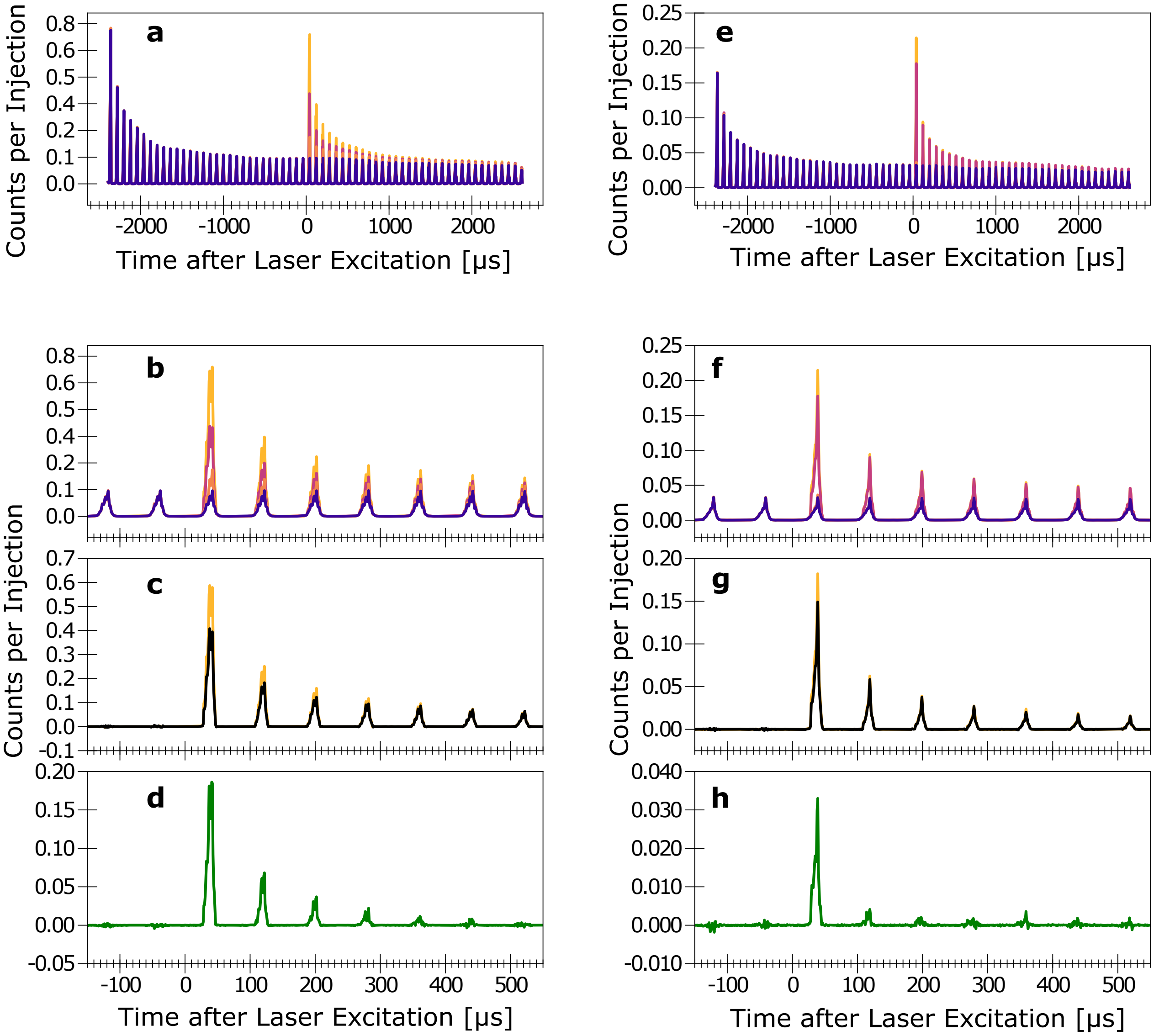

Supplementary Figure 1. Counts of fragments on the MCP detector as a function of storage time. Time zero is defined as the firing time of the pump laser. The data shown were accumulated for pump-probe delays larger than 700 fs for 580 nm probe, and for less than 700 fs for a 800 nm probe. Counts are generated only when an ion bunch is approaching the detector, hence peaks appear with time intervals defined by the revolution time (80 μs) in SAPHIRA. **a-d** 580 nm pump and 580 nm probe. **e-h** 580 nm pump and 800 nm probe. **a, b, e,** and **f** The blue trace was recorded without laser pulses where counts were solely due to unimolecular dissociation (hot ions from the source) or collisionally-induced fragmentation. Firing only the pump or probe laser, which might also lead to absorption and thus fragmentation depending on the photon wavelengths, yielded the orange and red traces respectively. Finally, the yellow traces show the recorded signal when both a pump and a probe pulse were fired. In **c** and **g**, the one-pulse signals (the sum of pump only and probe only with the background signal subtracted) are shown as the black traces. The green traces in **d** and **h** are the background corrected pump-probe signal, obtained when both a pump and a probe pulse were absorbed.

λ_{pump} (nm)	λ_{probe} (nm)	$t < 0$ τ_{short} (fs)	$t > 0$ τ_{short} (fs)	τ_{long} (ps)
570	800	–	587 ± 102	5.3 ± 2.3
580	400	493 ± 66	910 ± 294	3.0 ± 1.4
580	800	–	395 ± 42	3.7 ± 0.4
580	900	–	440 ± 110	4.1 ± 1.9
600	800	–	424 ± 121	2.5 ± 0.3
620	800	–	409 ± 136	2.6 ± 0.7
640	800	–	383 ± 47	7.6 ± 4.4

Supplementary Table 1. Exponential fit results Characteristic lifetimes obtained by fitting a double exponential decay function, or single exponential recovery function to individual spectra. Positive times corresponds to the situation where the pump pulse is applied before the probe pulse.

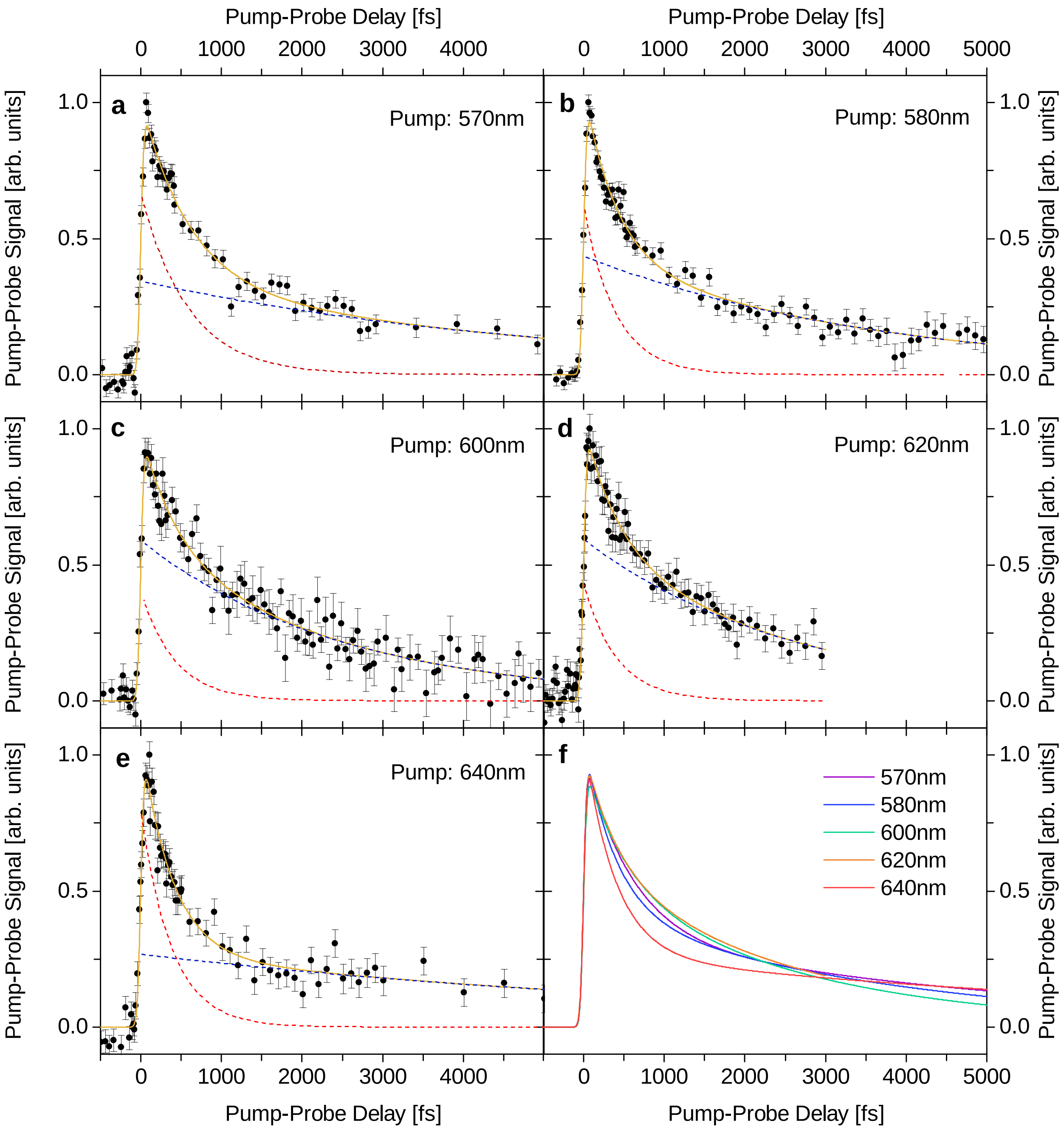

Supplementary Figure 2. Two-photon, pump-probe signal as a function of delay between pump and (fixed 800 nm) probe pulses. Pump pulses are in the spectral region of S_1. The full curves are from fits with two exponential functions folded with the 80 fs cross correlation in the experiment. Dashed lines indicate the components in the fits. All errorbars presented are one standard deviation (SD).

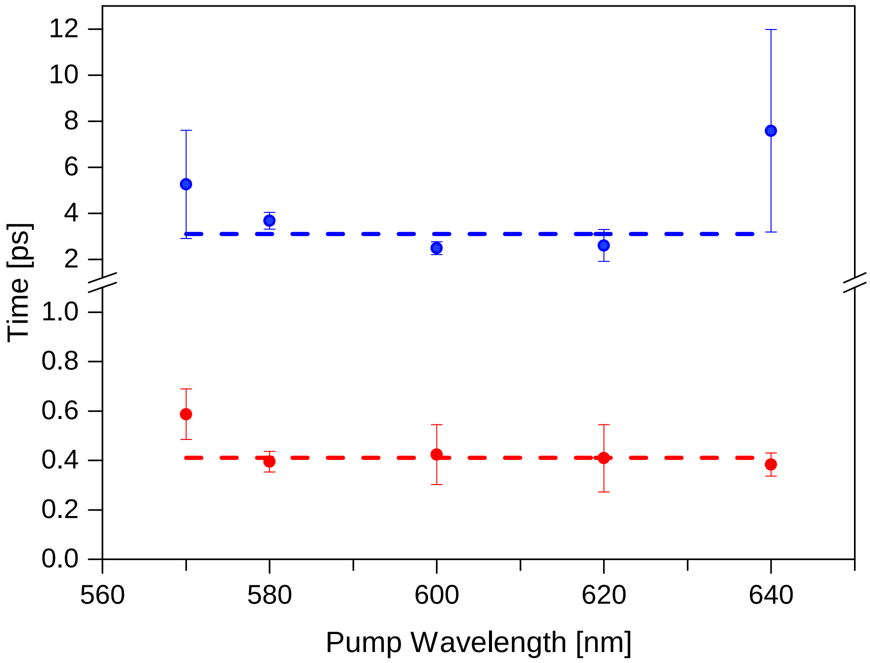

Supplementary Figure 3. Wavelength dependence of lifetimes. The short and long lifetimes as a function of the pump wavelength (800 nm probe wavelength), obtained from two-component fits to the excited-state decay (see data with fits in Supplementary Figure 2.). The dashed lines represent the average lifetimes for the data. Note that the short lifetime component of about 400 fs here represents an average of a two fast components - see Fig. 2 **a** in the main text. All errorbars presented are one standard deviation (SD).

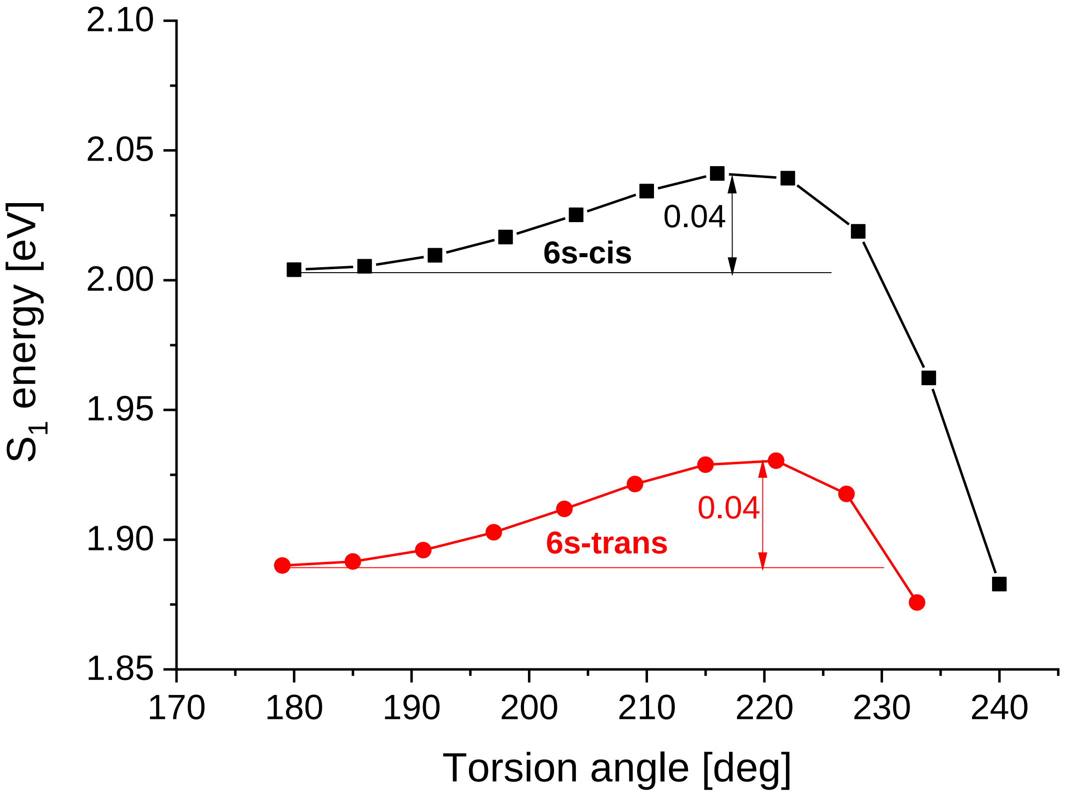

Supplementary Figure 4. Calculated potential energy curves along the isomerization coordinates in 6s-cis and 6s-trans rotomers of all-trans RPSB. Energies of the first excited state are shown with respect to the corresponding minima in the ground state. Note that the 6s-cis and 6s-trans rotomers have the same barrier heights that hinder isomerization in S_1 in all-trans RPSB. See also Fig. 4 in the main text.
